# O040. Migraineurs and self-consciousness of illness in a population of hospital workers

**DOI:** 10.1186/1129-2377-16-S1-A71

**Published:** 2015-09-28

**Authors:** Giovanna Viticchi, Lorenzo Falsetti, Laura Buratti, Andrea Plutino, Leandro Provinciali, Mauro Silvestrini, Marco Bartolini

**Affiliations:** Neurological Clinic, Marche Polytechnic University, Ancona, Italy; Internal and Subintensive Medicine, Ospedali Riuniti, Ancona, Italy

## Background

The World Health Organization has classified headache as the 14^th^ cause of disabling illnesses. Millions of people are affected by this pathology, especially during their working life. Surprisingly, subjects affected by headache, in particular by migraine, present a low degree of self-awareness of their pathology, and they do not usually consult any headache center[1]. For this reason migraineurs often go towards serious complications, such as chronicization or drug abuse. We investigated, in a selected population of workers, employees of the Ospedali Riuniti in Ancona, the number of people affected by headache and their awareness. We also investigated the drugs used to verify the use of a specific therapy for such a diffused pathology.

## Methods

We submitted all types of health workers (physicians, nurses, technicians, sanitary operators) to an anonymous questionnaire concerning the presence of headache and its characteristics. Particularly, we investigated if these subjects referred to their general practitioner or to a headache center for their symptoms. We also tried to understand the drugs employed by these people. The type of drugs and the category of the working activity were synthesized as two different ordinal variables. Difference in the distribution of the different drug categories was evaluated with χ^2^ test. Statistics was performed with SPSS 13.0 for Windows systems.

## Results

We enrolled 1,700 consecutive subjects: 18.1% of the population (308 patients) resulted affected by migraine. Only a minimum part of these patients had consulted a headache center in their life. Subjects tended not to take any drugs for their acute attack of headache, or took significantly more non-steroidal anti-inflammatory drugs (NSAIDs) in respect to triptans. Distribution of the use of the drugs resulted significantly different (p < 0.0001) with χ^2^ test.

## Conclusions

Migraineurs, also in presence of more than one attack in their life, typically showed low self-awareness about their condition and usually did not refer to a specialistic headache center. Consequently, the use of specific molecules, such as triptans, presented a very low diffusion. These results reflect international literature, but on the other hand, underline a very unsatisfactory knowledge about migraine and its possible consequences[2, 3]. Moreover, these data are especially worrisome because they are representative of a hospital population. Better education and awareness about such a prevalent and disabling pathology and its management should be favored.

Written informed consent to publication was obtained from the patient(s).
